# RETRACTION: Association Between Vitamin D Deficiency and Diabetic Foot Ulcer Wound in Diabetic Subjects: A Meta‐Analysis

**DOI:** 10.1111/iwj.70427

**Published:** 2025-03-26

## Abstract

**RETRACTION:** LinJ. , MoX. , YangY. , TangC. , and ChenJ. , “Association Between Vitamin D Deficiency and Diabetic Foot Ulcer Wound in Diabetic Subjects: A Meta‐Analysis,” International Wound Journal 20, no. 1 (2023): 55–62, 10.1111/iwj.13836.35567425 PMC9797924

The above article, published online on 14 May 2022, in Wiley Online Library (http://onlinelibrary.wiley.com/), has been retracted by agreement between the journal Editor in Chief, Professor Keith Harding; and John Wiley & Sons Ltd. Following an investigation by the publisher, all parties have concluded that this article was accepted solely on the basis of a compromised peer review process. The editors have therefore decided to retract the article. The authors did not respond to our notice regarding the retraction.

**RETRACTION:** J. Lin , X. Mo , Y. Yang , C. Tang , and J. Chen , “Association Between Vitamin D Deficiency and Diabetic Foot Ulcer Wound in Diabetic Subjects: A Meta‐Analysis,” International Wound Journal 20, no. 1 (2023): 55–62, 10.1111/iwj.13836.35567425 PMC9797924

The above article, published online on 14 May 2022, in Wiley Online Library (http://onlinelibrary.wiley.com/), has been retracted by agreement between the journal Editor in Chief, Professor Keith Harding; and John Wiley & Sons Ltd. Following an investigation by the publisher, all parties have concluded that this article was accepted solely on the basis of a compromised peer review process. The editors have therefore decided to retract the article. The authors did not respond to our notice regarding the retraction.

